# Long Non-Coding RNA HAND2-AS1 Acts as a Tumor Suppressor in High-Grade Serous Ovarian Carcinoma

**DOI:** 10.3390/ijms21114059

**Published:** 2020-06-05

**Authors:** Priyanka Gokulnath, Tiziana de Cristofaro, Ichcha Manipur, Tina Di Palma, Amata Amy Soriano, Mario Rosario Guarracino, Mariastella Zannini

**Affiliations:** 1 IEOS–Institute of Experimental Endocrinology and Oncology G. Salvatore, National Research Council, via S. Pansini 5, 80131 Napoli, Italy; priyanka.gokulnath@gmail.com (P.G.); t.decristofaro@ieos.cnr.it (T.d.C.); t.dipalma@ieos.cnr.it (T.D.P.); amata.soriano@libero.it (A.A.S.); 2 Department of Environmental, Biological and Pharmaceutical Sciences and Technologies, University of Campania Luigi Vanvitelli, 81100 Caserta, Italy; ichcha.manipur@icar.cnr.it; 3 High Performance Computing and Networking Institute, National Research Council, via P. Castellino 111, 80131 Napoli, Italy; mario.guarracino@cnr.it; 4 HSE–National Research University Higher School of Economics, LATNA Laboratory, 603155 Nizhny Novgorod, Russia

**Keywords:** long non-coding RNA, HAND2-AS1, high grade serous ovarian carcinoma, fallopian tube, tumor suppressor, mRNA network, ceRNA network

## Abstract

Long non-coding RNAs (lncRNAs) are increasingly being identified as crucial regulators in pathologies like cancer. High-grade serous ovarian carcinoma (HGSC) is the most common subtype of ovarian cancer (OC), one of the most lethal gynecological malignancies. LncRNAs, especially in cancers such as HGSC, could play a valuable role in diagnosis and even therapy. From RNA-sequencing analysis performed between an OC cell line, SKOV3, and a Fallopian Tube (FT) cell line, FT194, an important long non-coding RNA, HAND2 Anti sense RNA 1 (HAND2-AS1), was observed to be significantly downregulated in OCs when compared to FT. Its downregulation in HGSC was validated in different datasets and in a panel of HGSC cell lines. Furthermore, this study shows that the downregulation of HAND2-AS1 is caused by promoter hypermethylation in HGSC and behaves as a tumor suppressor in HGSC cell lines. Since therapeutic relevance is of key importance in HGSC research, for the first time, HAND2-AS1 upregulation was demonstrated to be one of the mechanisms through which HDAC inhibitor Panobinostat could be used in a strategy to increase HGSC cells’ sensitivity to chemotherapeutic agents currently used in clinical trials. To unravel the mechanism by which HAND2-AS1 exerts its role, an in silico mRNA network was constructed using mRNAs whose expressions were positively and negatively correlated with this lncRNA in HGSC. Finally, a putative ceRNA network with possible miRNA targets of HAND2-AS1 and their mRNA targets was constructed, and the enriched Gene Ontology (GO) biological processes and Kyoto Encyclopedia of Genes and Genomes (KEGG) pathways were identified.

## 1. Introduction

Long non-coding RNAs (lncRNAs) are non-translated RNAs, 200–10,000 nucleotides in size, that act as masters of genome regulation [1]. Their ability to form secondary structures, localize in the nucleus and cytoplasm in a cell, and even survive in extracellular fluid coupled together with their ability to bind to both proteins and nucleic acids have endowed them a lot of control in most cellular processes [2]. Research on lncRNAs is a growing field in molecular biology, keeping in mind their vast milieu and their diverse roles in physiology and pathology, especially in cancer [3]. They have been attributed a role in bringing about every cancer hallmark and have an important contribution towards almost all regulatory process at a cellular level [4]. 

High-grade serous ovarian cancer (HGSC) is the most incident and lethal subtype of ovarian cancer (OC) [5], which in turn is the most common cause of mortality in women due to gynecological malignancy [6]. This is a very aggressive cancer subtype that is almost always detected after intraperitoneal spread due to the lack of early markers or unique diagnostic symptoms [7]. Though this cancer was thought to be de novo or sporadic, recent research has attributed the fallopian tube (FT) to be the initial site of tumorigenesis, evidenced by serous tubal intraepithelial carcinomic lesions in more than 60% of HGSC patients [7]. Despite decades of research, there has been no considerable improvement in the life expectancy of HGSC patients. Therefore, the identification of prognostic or diagnostic lncRNA markers for HGSC can be of critical value. Several lncRNAs, such as H19, HOTAIR, MALAT1, PVT1, and UCA1, have been previously reported to act as diagnostic lncRNAs in OC [8]. In this article, we add HAND2-AS1 to this growing list of lncRNAs with an important role specifically in HGSC.

HAND2-AS1/DEIN/Uph was first reported to be expressed in cardiac muscle cells, where it was demonstrated to have an important role in regulating cardiac development by controlling the expression of the coding protein HAND2 [9] and also share the promoter, which is bidirectional [9,10]. It was even observed to be a prognostic marker in cardiac myopathies [11]. However, with respect to cancer, its role as a tumor suppressor was first reported in endometroid endometrial cancer [12]. Subsequently, HAND2-AS1 was observed to play an essential role in many cancers, such as osteosarcoma [13], colorectal cancer [14], lung cancer [15], leukemia [16], esophageal cancer [17], and more recently, even in ovarian cancer [18]. In most of the cases, HAND2-AS1 acts as a competing endogenous RNA with a tumor-suppressive role to regulate the expression of a microRNA and in turn affecting the target mRNA expression. Here, we show the role of HAND2-AS1 in HGSC, the worst subtype of OC, and identify a translational approach for the better management of HGSC patients.

## 2. Results

### 2.1. HAND2-AS1 as an Important Long Non-Coding RNA (lncRNA) in High-Grade Serous Carcinoma (HGSC)

**Figure 1:**
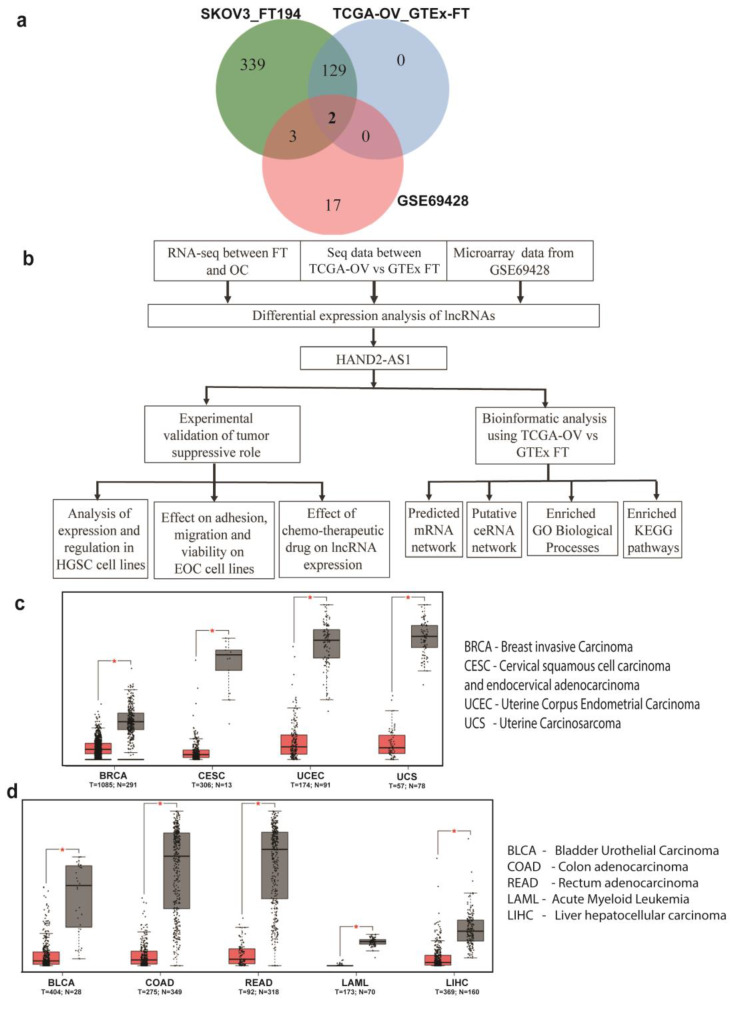
(**a**) Venn diagram showing the distribution of significantly differentially expressed long non-coding RNAs from transcriptome analysis between high-grade serous ovarian carcinoma (HGSC) and fallopian tube (FT) samples; (**b**) Schematic representation of the flow of analysis used to analyze HAND2 Anti sense RNA 1 (HAND2-AS1) in this study; Expression of HAND2-AS1 in (**c**) gynecological cancers; and (**d**) other cancers obtained from The Cancer Genome Atlas (TCGA) using Gene Expression Profiling Interactive Analysis (GEPIA) (between tumor (red) and normal (grey) tissue).

To better understand the mechanisms by which long non-coding RNAs (lncRNAs) are involved in the development of HGSC, an RNA-seq experiment was performed between an ovarian cancer cell line (SKOV3) and a fallopian tube cell line (FT-194). In this experiment, several significantly downregulated non-coding RNAs were identified [19]. Of these, lncRNA HAND2-AS1 was one of the topmost significantly downregulated RNAs and was reported to behave as a tumor suppressor in several other cancers [12,13,14,15,16,17,18,20]. To further confirm the role of HAND2-AS1 in HGSC originating from the fallopian tube, two other datasets between HGSC and FT were validated. The first was the comparative transcriptome analysis between ovarian cancer and FT tissue controls obtained from The Cancer Genome Atlas—Ovarian Cancer (TCGA-OV) and Genotype Tissue Expression—Fallopian Tube (GTEx-FT) samples [19]. The second was an independent study [21] identified from the Gene Expression Omnibus (GEO) dataset (GSE69428) with 10 HGSC patient samples and 10 normal FT samples. The differentially expressed lncRNAs from the three different studies are listed in Supplementary Table S1, and only two lncRNAs, XIST and HAND2-AS, were observed to be common from the different studies as shown in Figure 1a. The flow chart of the analysis of HAND2-AS1 in HGSC originating from FT with respect to the experimental and bioinformatics approach is shown in Figure 1b.

Therefore, to better verify its role in cancer, the expression of HAND2-AS1 was examined in other cancers, using the webtool Gene Expression Profiling Interactive Analysis (GEPIA) [22]. This is illustrated using the box plots shown in Figure 1c,d. Figure 1c shows that HAND2-AS1 is consistently and significantly downregulated in gynecological cancers and Figure 1d demonstrates other cancers that downregulate this lncRNA significantly.

### 2.2. HAND2-AS1 Expression Is Consistently Downregulated in Ovarian Cancer Cell Lines Possibly Due to Promoter Hypermethylation

**Figure 2:**
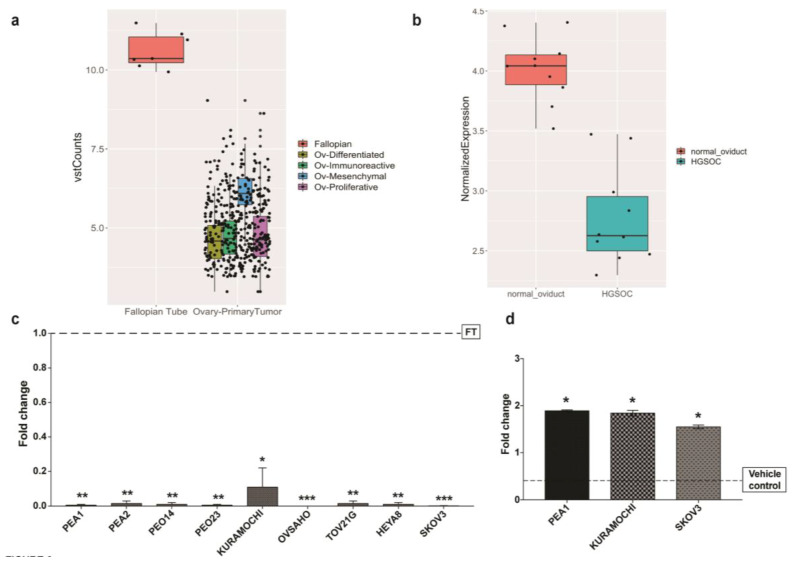
Analysis of long non coding RNA (lncRNA) HAND2-AS1 expression plotted as box plots between HGSC and FT tissues obtained from (**a**) The Cancer Genome Atlas–Ovarian Cancer (TCGA-OV) and Genotype Tissue Expression (GTEx-FT); (**b**) GSE69428; (**c**) Expression of HAND2-AS1 in several ovarian cancer (OC) cell lines with control as primary FT cells (FT); (**d**) Expression of HAND2-AS1 in KURAMOCHI, PEA1, and SKOV3 cells after 5-Aza-2’-deoxycytidine (5-AZA) treatment along with the corresponding vehicle control. Values from experiments (n = 2) are expressed as means ± SD from real-time PCR performed in duplicates normalized using house-keeping gene Abelson (ABL) expression. Student’s t-test (two-tailed) was used to calculate the *p*-value between control and test groups with the cutoff as *p* ≤ 0.05, wherein asterisks denote significance. * *p* < 0.05, ** *p* < 0.01, *** *p* < 0.001.

Figure 2a,b show the expression of HAND2-AS1 between HGSC vs. FT from TCGA-OV/GTEx-FT and GSE69428 datasets, respectively. To further expand our validation in OC, we examined the expression of HAND2-AS1 (Figure 2c) using qPCR in several ovarian cancer (OC) cell lines with respect to primary human fallopian tube epithelial cells (FT) taken as normal. The panel of OC cell lines consisted of PEA1, PEA2, PEO14, PEO23, OVSAHO, KURAMOCHI, HeyA8, SKOV3, and TOV21G cell lines. HAND2-AS1 is indeed downregulated in all the ovarian cancer cell lines with respect to the primary fallopian tube cells, suggesting a putative role in OC, specifically in HGSC. 

HAND2-AS1 is known to undergo hypermethylation in other cancers [12,13] and further confirmed in endometrial carcinoma [12]. Additionally, in a microarray study conducted [23] to identify genome-wide de novo methylation analysis in ovarian cancer, the HAND2 promoter, which is also shared with HAND2-AS1 [11], was one of the sites identified. Though these are indirect conclusions, to understand the HAND2 promoter methylation status in ovarian cancer, we modeled the analysis as performed in the above study [12] and conducted an in silico analysis of the HAND2-AS1 promoter (Supplementary Figure S1a), which showed two CpG islands of which CpG 120 in the promoter region could impede HAND2-AS1 expression when methylated. The methylation status of HAND2-AS1 promoter was further verified using an online software DiseaseMeth 2.0 [24], which showed that HAND2-AS1 is hypermethylated in several cancers, such as bladder carcinoma, lung adenocarcinoma, pancreatic adenocarcinoma, head and neck cancers, colon carcinoma, and anal carcinoma (Supplementary Figure S1b). Based on these observations, we hypothesized that HAND2-AS1 could be epigenetically downregulated in HGSC due to promoter hypermethylation. To understand whether this was the case, we treated three OC cell lines, PEA1, KURAMOCHI, and SKOV3 (PEA1 and KURAMOCHI are HGSC cell lines) with the demethylation inhibitor 5-Aza-2’-deoxycytidine (5-AZA) for 72 h and checked the expression of HAND2-AS1 before and after the treatment using qPCR. As observed in Figure 2d, there is an increase in the expression of HAND2-AS1 after the inhibition of demethylating agents. This suggests that the downregulation of HAND2-AS1 observed in HGSC cell lines is due to promoter hypermethylation.

### 2.3. Expression of HAND2-AS1 in Ovarian Cancer Cells Decreases Their Adhesion to Extracellular Matrix, Migration, and Viability

**Figure 3:**
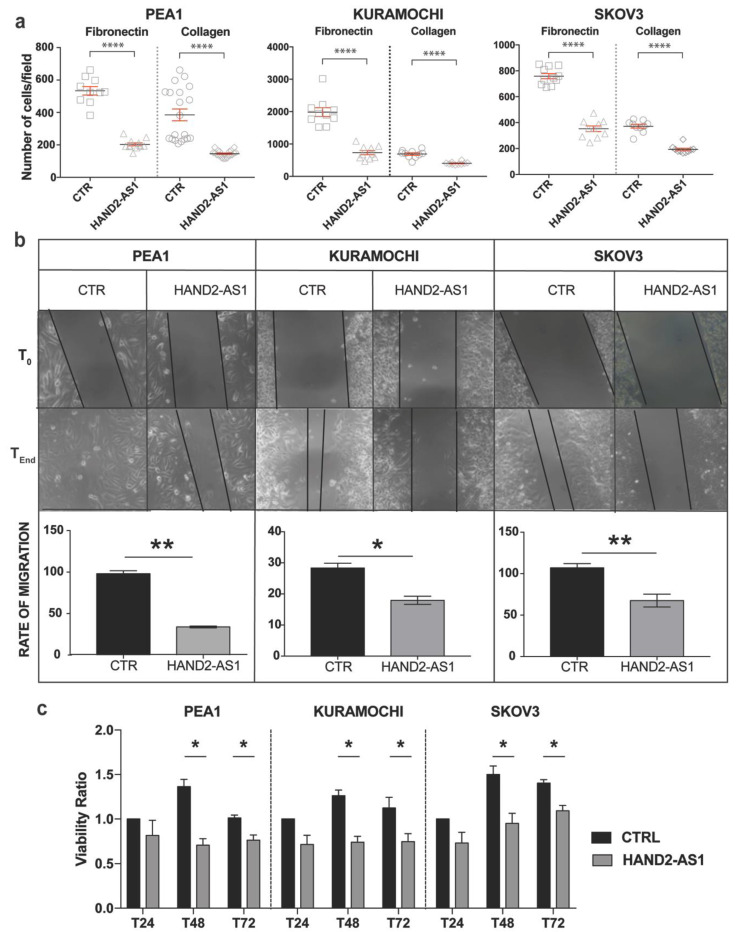
Effect of HAND2-AS1 expression on physiological properties in three cell lines–after PEA1, KURAMOCHI, and SKOV3 cells after transfection with HAND2-AS1 and control expression vectors: (**a**) adhesion to fibronectin and collagen I-coated ECM substrates quantified in terms of the number of attached cells per field, (**b**) migration denoted by representative images and quantitative representation showing cumulative rates of migration, and (**c**) the viability ratio between the absorbance of control and HAND2-AS1 vector obtained at 24, 48, and 72 h represented as T24, T48, and T72. Values from experiments (n = 3) are expressed as means ± SD normalized using control vector-treated samples. For the adhesion assay, the Mann–Whitney U test was used to assess significance with a *p*-value cutoff at *p* ≤ 0.05 with asterisks denoting significance as **** *p* ≤ 0.0001. Significance in the migration assay and viability assay was assessed by calculating the *p*-value between control and HAND2-AS1-transfected samples using Paired and Student’s t-test (two-tailed), respectively, with the cutoff as *p* ≤ 0.05 wherein asterisks denote significance. ns not significant, * *p* < 0.05, ** *p* < 0.01.

To understand whether HAND2-AS1 has a role consistent with that of a tumor suppressor, its physiological functions were explored after its expression using plasmid-derived HAND2-AS1 expression vector in OC cell lines (Supplementary Figure S2). Therefore, we transfected these cell lines with HAND2-AS1 and control vector and then chose to assess the adhering capacity of the transfected cells by examining their ability to bind to fibronectin- or collagen-coated coverslips mimicking the extra cellular matrix (ECM). HAND2-AS1 expression was observed to decrease the adhesion of HGSC cell lines to ECM (Figure 3a). Next, the effect of HAND2-AS1 expression on the migratory abilities of HGSC cell lines was evaluated through the wound healing assay. Figure 3b demonstrates the reduction in the migratory abilities using the rate of migration, which decreased in all three cell lines by HAND2-AS1, accompanied by representative images obtained at T_0_ and T_end_. 

Further, we checked the viability of the HGSC cell lines after transfection with control and HAND2-AS1 vectors using the MTT assay. Figure 3c demonstrates that the expression of HAND2-AS1 is capable of decreasing the viability of HGSC cell lines though the reason for decreased viability (either due to a decrease in cell proliferation or increased cell death) should be investigated further.

### 2.4. HAND2-AS1 Expression Is Rescued after Treatment with HDAC Inhibitor

HAND2-AS1 has previously been shown to have a role as a prognostic/diagnostic biomarker in lung cancer by its detection in blood [15]. Though the tumor-suppressive role of HAND2-AS1 has been demonstrated in HGSC cell lines, it was necessary to precisely understand if HAND2-AS1 has a putative therapeutic role in OC.

**Figure 4:**
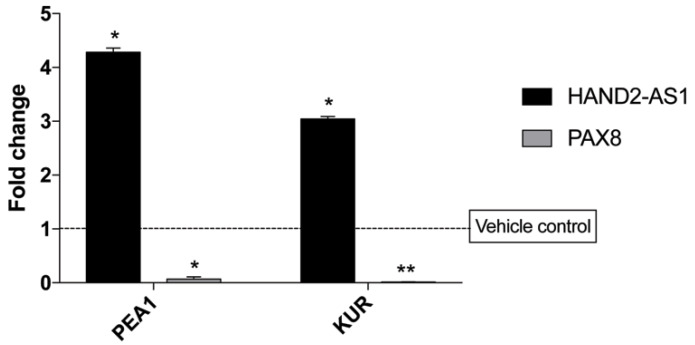
Expression of HAND2-AS1 and PAX8 in PEA1 and KURAMOCHI cells upon 48 h of treatment with the HDAC inhibitor Panobinostat and the corresponding vehicle control. Values from experiments (n = 2) are expressed as means ± SD from real-time PCR performed in duplicates normalized using the house-keeping gene Abelson (ABL) expression. *p*-value was calculated between control and test groups using Student’s t-test (two-tailed) with the cutoff as *p* ≤ 0.05 wherein asterisks denote significance. * *p* < 0.05, ** *p* < 0.01.

Numerous small molecules functioning as HDAC inhibitors are being considered for clinical use [25]. Recently, one such HDAC inhibitor, Panobinostat, was demonstrated to act in synergy with cisplatin to increase the efficacy of chemotherapy [26,27]. Panobinostat has been reported to be very effective in OC specifically by causing the downregulation of PAX8 expression, which in turn is a crucial OC promoting transcription factor [28]. From the work described by Anderson et al. [9], HAND2-AS1 expression has been observed to be governed by the histone acetylation status. The rescue of HAND2-AS1 might provide a good therapeutic approach given its tumor-suppressive role. It is quite interesting to observe that the treatment of Panobinostat on two HGSC cell lines, PEA1 and KURAMOCHI, not only caused a decrease in the expression of PAX8 (Figure 4) but a concomitant increase in the expression of lncRNA HAND2-AS1. The SKOV3 cell line was also treated with Panobinostat and an increase in HAND2-AS1 expression was observed, while there was no decrease in PAX8 expression (data not shown).

### 2.5. Identification of the HAND2-AS1-Associated mRNA and ceRNA Network along with Enriched Gene Ontology Biological Processes and Pathways in HGSC

**Figure 5:**
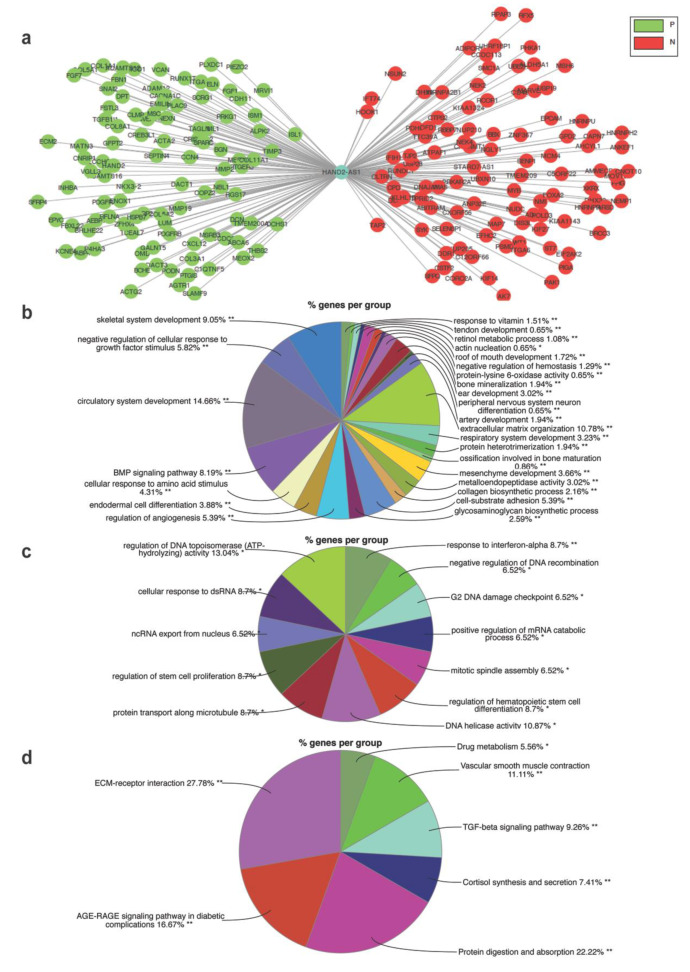
(**a**) Proposed mRNA network constructed bioinformatically for HAND2-AS1 with putative target mRNAs positively and negatively correlated with HAND2-AS1 (cBioportal) in HGSC; All Gene Ontology (GO) biological process terms enriched by (**b**) positively correlated mRNAs and (**c**) negatively correlated mRNAs with HAND2-AS1; (**d**) all Kyoto Encyclopedia of Genes and Genomes (KEGG) pathways enriched by positively correlated mRNA with HAND2-AS1 in HGSC obtained using Cytoscape.

The previous experiments established that HAND2-AS1 acts as a tumor suppressor in OC, specifically in HGSC. Further, to unravel the mechanism by which HAND2-AS1 can bring about a tumor-suppressive effect, an mRNA network (Figure 5a) using genes positively and negatively correlated with HAND2-AS1 (Supplementary Table S2) was constructed after examining their expression in HGSC samples (TCGA-OV) in cBioportal [29,30]. The Gene Ontology (GO) biological process terms enriched from these positively (Supplementary Table S3) and negatively correlated mRNAs (Supplementary Table S4) were visualized using Cytoscape [31,32] and are shown in Figure 5b,c, respectively. The enriched Kyoto Encyclopedia of Genes and Genomes (KEGG) terms obtained from the mRNAs positively correlated with HAND2-AS1(Supplementary Table S5) are shown in Figure 5d. 

**Figure 6:**
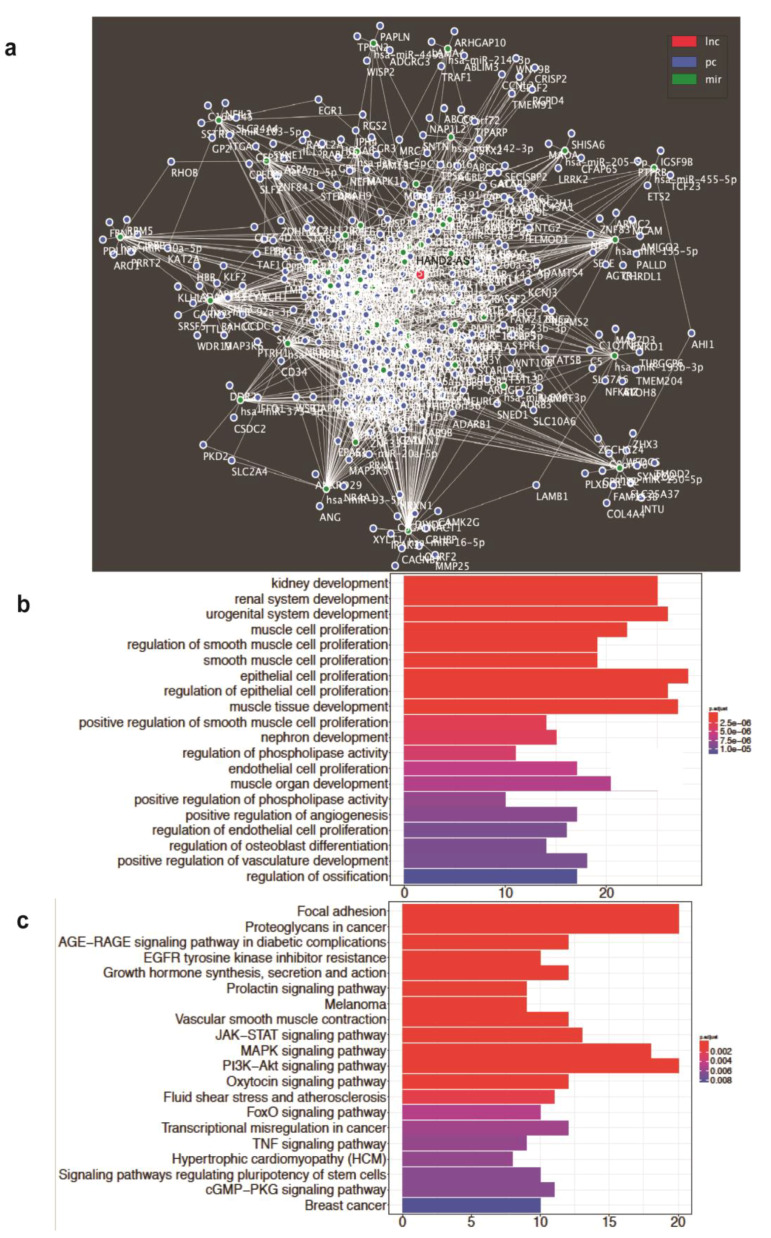
(**a**) Proposed competing endogenous RNA (ceRNA) network constructed bioinformatically for HAND2-AS1 with its target miRNAs (predicted using webtool miRTarBase) and their putative mRNA targets in HGSC (predicted using the webtool Diana); (**b**) All GO biological process terms; and (**c**) all KEGG pathways enriched by the HAND2-AS1 competing endogenous RNA (ceRNA) activity in HGSC obtained using the GDCRNATools R-package.

HAND2-AS1 behaves like a sponge for miRNAs in other cancers [14,16,17]. Recently, its role as a competing endogenous RNA (ceRNA) has been described in OC for miR-340-5p, but the study was performed considering ovary tissue as the control [18]. Therefore, we aimed to better understand the mechanism by which HAND2-AS1 can act as a tumor suppressor in HGSC originating from FT. Towards this, all putative miRNAs (Supplementary Table S6) differentially regulated between HGSC and FT, which show binding to HAND2-AS1, were consolidated, and a ceRNA network was constructed, validated by the patient data from TCGA-OV using the GDCRNATools R package [33]. This network is shown in Figure 6a and it indicates the possible putative miRNAs and their downstream mRNA targets that are experimentally modified by HAND2-AS1 in HGSC with respect to FT. Further, all the enriched GO biological processes and KEGG pathways were identified from this ceRNA network are shown in Figure 6b,c. However, they have to be experimentally validated and further investigated in the future to determine the exact mechanism of HAND2-AS1 in HGSC originating from FT.

## 3. Discussion

HGSC is the worst subtype of OC [34], with relapse after chemotherapy being the primary cause of death in patients [35]. Despite decades of research, the percentage of lethality of patients has not seen a significant improvement as the 5-year survival rate remains similar [21]. This is because chemotherapeutic resistance has been the most critical challenge that affects mortality in HGSC. Therefore, any new research that identifies key players that can act either as biomarkers for the detection or prognostic markers to predict therapeutic efficacy is highly welcome in the case of HGSC. Preliminary steps in this direction have been taken, as demonstrated by our work done in this paper. 

Towards the aim of finding an important biomarker for HGSC, we analyzed three different datasets, RNA sequencing (SKOV3 vs. FT194), TCGA-OV vs. GTEx-FT, and GSE69428. Among the three datasets, only XIST and HAND2-AS1 were observed to be common in all. While XIST has been thoroughly studied in the context of malignancy, HAND2-AS1’s role is relatively new in ovarian cancer, which prompted us to pursue investigations regarding this lncRNA.

HAND2-AS1, an antisense lncRNA for the HAND2 coding gene, has been incredibly useful in the prognosis of cardiac myopathies [11]. This lncRNA has also been detected as a serum biomarker in osteosarcoma [36], and its potential biomarker role in HGSC should be thoroughly investigated, given the fact that HGSC has no early detection markers. Therefore, we consolidated three studies comparing HGSC and control FT samples to identify HAND2-AS1 as an important lncRNA in HGSC. Since this lncRNA is consistently downregulated in HGSC and several other malignancies, including gynecological ones, its role towards cancer has become more interesting and relevant.

The role of HAND2-AS1 as a tumor suppressor has been previously reported in OC [18]. However, the current study is unique primarily because it gives the scope of lncRNA HAND2-AS1 in HGSC therapy. This is highly significant because an increase in the activity of histone deacetylases (HDAC) is usually associated with chemoresistance, and very few drugs offer promising therapy with respect to HGSC [27]. In this respect, Panobinostat, a HDAC inhibitor, is able to effectively combat OC growth when given in combination with cisplatin [26]. When we treated HGSC cell lines with Panobinostat, we observed a decrease in the expression of PAX8, an important protumorigenic marker of HGSC, as previously reported [28]. We propose another mechanism by which the drug Panobinostat brings about HGSC cell death: Through HAND2-AS1 expression that possibly activates tumor-suppressive pathways. Though Panobinostat is a HDAC inhibitor, the upregulation of HAND2-AS1 expression is one of the ways through which the efficacy of HGSC therapy can be assessed post chemotherapy to evaluate resistance and understand its prognostic value. This study also demonstrates consistently the tumor-suppressive role of HAND2-AS1 in OC, specifically in HGSC cell lines.

Further, this study aimed to gain better insights into the HAND2-AS1 mechanism. For the first time, all the mRNAs that could be regulated by HAND2-AS1 in HGSC were identified using bioinformatic methods. This approach revealed the enriched GO BPs and pathways regulated by the mRNAs whose expression, in turn, is possibly modulated by HAND2-AS1 in HGSC. Some of the key observations that could direct the future focus of HAND2-AS1 research is the fact that this lncRNA positively regulates processes, such as extracellular matrix organization and tube morphogenesis, suggesting that it could be involved in the maintenance of normal FT tissue. In addition, its negative regulation of processes, such as stem cell proliferation and regulation of DNA recombination, also lays a rough basis for its tumor-suppressive role in HGSC.

HAND2-AS1 is known to behave as a competing endogenous RNA (ceRNA) [14,16,17] and this study has generated a putative HAND2-AS1 ceRNA network that provides insights towards the possible miRNA and mRNA players regulated by HAND2-AS1. Further, all the GO BP and pathways enriched by this network are also indicated. The effect on KEGG pathways, such as focal adhesion and proteoglycans, in cancer suggest that HAND2-AS1 could have a crucial role in adhesion and migratory properties as validated by our experimental assays in this study.

**Figure 7:**
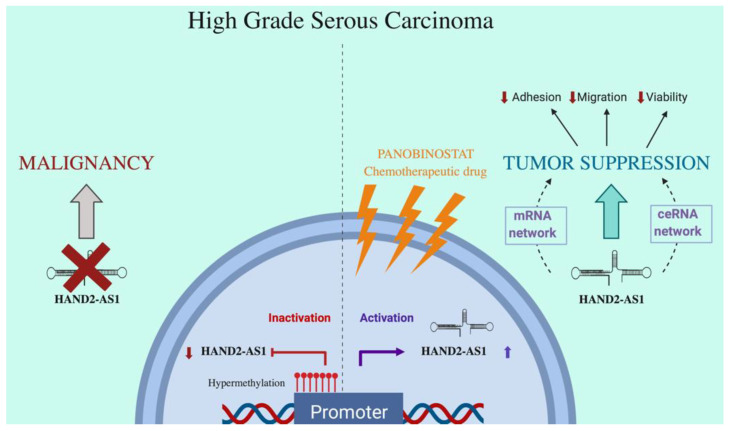
Schematic summary of the role of HAND2-AS1 in HGSC. 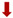
—denotes downregulation, 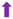
—denotes upregulation.

Taking the two approaches together, lncRNA HAND2-AS1 are predicted to be involved in the maintenance of homeostasis in normal tissue while its downregulation appears to be sustained in the malignant phenotype. Even though both the bioinformatics analyses performed in this study provided a peek into the function of HAND2-AS1 in HGSC, a more thorough experimental validation is undoubtedly required to chalk out its complete mechanism. Coupled together with an increase in the expression of this lncRNA upon treatment with Panobinostat, it certainly offers a promising role for HAND2-AS1 as a prognostic marker in HGSC. All the key findings of this study are clearly summarized in Figure 7.

Further, to increase the diagnostic efficacy of HGSC using lncRNAs, a bioinformatic approach must be taken up to identify a possible diagnostic signature, using a sufficient number of HGSC and associated normal samples, that could be detected early enough in HGSC patients. It is only prudent to mention that this field is still at its infancy and will be considerably benefitted by parallel advancement of next-generation techniques and bioinformatics approaches along with molecular biological techniques.

## 4. Materials and Methods

### 4.1. Differential Analysis of Coding and Long Non-Coding RNAs

The RNA samples extracted from SKOV-3 (ovarian cancer cell line) and FT-194 (fallopian tube epithelial cell line) were sequenced by the Illumina HiSeq 1500 platform using a resolution at 100 base-pairs with paired-end reads. The analysis was performed using the RAP (RNA-Seq Analysis Pipeline) available on https://bioinformatics.cineca.it/97. The sequences’ quality check, mapping, transcriptome assembly, and differential expression analyses were performed using the default parameters on RAP. An alpha level of 0.05 was used for all the statistical tests.

RNA sequencing gene counts data of 430 samples of ovarian serous cystadenocarcinoma from the TCGA-OV project, and 7 healthy Fallopian tissue samples from GTEx were downloaded from the recount2 website (https://jhubiostatistics.shinyapps.io/recount/). They were processed by the methods previously described in [19].

Microarray expression data for 10 samples of high-grade serous ovarian cancer and 10 normal oviduct samples were obtained from the NCBI Gene Expression Omnibus database with the accession code GSE69428. The limma package was used to extract differentially expressed genes with log2 fold-change ≥ |1| and an adjusted *p*-value ≤ 0.05 between the HGSC and the normal oviduct samples.

### 4.2. Expression of HAND2-AS1 in Various Cancers

Differential expression analysis was performed between various cancers and their matched normal samples and was visualized using boxplots with the online tool Gene Expression Profiling Interactive Analysis (GEPIA) [22] (http://gepia.cancer-pku.cn) by setting log2 fold change ≥ |1| and *p*-value ≤ 0.01. The expression between the normal samples (grey) from TCGA and GTEx and tumor samples (red) from TCGA were plotted in the log scale.

### 4.3. Venn Diagrams

All Venn diagrams were generated using the jVenn tool, which is available online [37] (http://jvenn.toulouse.inra.fr/app/index.html).

### 4.4. Cell Lines and Culture Methods

High-grade serous ovarian cancer cell lines PEA1, PEA2, PEO14, and PEO23 were purchased from Sigma-Aldrich (St. Louis, MO, USA) and were grown in RPMI-1640 medium supplemented with 10% fetal bovine serum, 2 mM glutamine, 2 mM sodium pyruvate, and 1% penicillin/streptomycin (Euroclone S.P.S., Pero, Italy). High-grade serous ovarian cancer cell lines KURAMOCHI and OVSAHO were obtained from the Japanese Collection of Research Bioresources Cell Bank (JCRB). The human ovarian cancer cell lines SKOV3, HeyA8 (High-grade serous ovarian cancer cell line), and TOV21G were provided by the CEINGE Cell Culture Facility (Naples, Italy). All these cell lines were maintained in RPMI-1640 medium supplemented with 10% fetal bovine serum and 1% penicillin/streptomycin (Euroclone S.P.S., Pero, Italy). The immortalized fallopian tube secretory epithelial cell line FT194 was provided by Dr. R. Drapkin (Boston, MA, USA). This cell line was grown in DMEM-F12 medium (Euroclone S.P.S., Pero, Italy) supplemented with 2% Ultroser G serum (PALL, Cergy-Saint-Christophe, France) and 1% penicillin/streptomycin. Primary human fallopian tube secretory epithelial cells were provided by Dr. U. Cavallaro (Milan, Italy) and was grown in appropriate medium [38].

### 4.5. Treatment with AZA

SKOV3, KURAMOCHI, and PEA1 cell lines were plated on the previous day and treated every 24 h with 10 µM of 5’-AZA (Sigma-Aldrich, St. Louis, MO, USA) and harvested after 72 h for RNA extraction.

### 4.6. Plasmid Preparation

The sequence of lncRNA HAND2-AS1 transcript (NCBI Reference sequence–NR_003679.2) was amplified by PCR from the FT194 cell line and constructed into pCDNA3.1 vector (Invitrogen, Carlsbad, CA, USA). The cloned vector was verified using sequencing provided by Eurofins Genomics service (Germany).

For migration and adhesion assays, KURAMOCHI, SKOV3, and PEA1 cell lines were transfected using Lipofectamine 3000 (Invitrogen, Carlsbad, CA, USA) with control vector (pCDNA3.1) or HAND2-AS1 using the manufacturer’s protocol and harvested for RNA extraction 48 h after transfection. 

For mRNA analysis, PEA1, KURAMOCHI, and SKOV3 cell lines were transfected using Lipofectamine 3000 (Invitrogen, Carlsbad, CA, USA) with control vector (pCDNA3.1) or HAND2-AS1 using the manufacturer’s protocol and harvested for RNA extraction 24 h after transfection. 

### 4.7. RNA, cDNA, and qRT-PCR

Total RNA was extracted using the RNeasy Mini kit (Qiagen, Hilden, Germany). The cDNA was synthesized using the iScript cDNA Synthesis kit (BIORAD, Hercules, CA, USA). Real-time qPCR analysis was performed using the IQTM SYBR Green PCR Master Mix (BIORAD, Hercules, CA, USA) in a CFX96 Real-Time PCR Detection System (BIORAD, Hercules, CA, USA) for the following genes using gene-specific primers:

ABL fwd 5′-TGGAGATAACACTCTAAGCATAACTAAAGG-3′
ABL rev 5′-GATGTAGTTGCTTGGGACCCA-3′
HAND2-AS1 fwd 5′-GTCTACGACTGCCTGTGACT-3′
HAND2-AS1 rev 5′-TGTCCCATATACAAGAATCGACA-3′
PAX8 fwd 5′-CCCTTCCAACACGCCACT-3′
PAX8 rev 5′-CTGCTTTATGGCGAAGGGTG-3′

For each gene, values are means ± SD of three independent experiments, normalized by the expression of housekeeping genes. To calculate the relative expression levels, we used the 2-ΔΔCT method [39].

### 4.8. Migration Assay

Migration assays were performed using Ibidi cell migration technology (Ibidi, Martinsried, Germany). SKOV3, KURAMOCHI, and PEA1 cell lines were transfected with HAND2-AS1 and control vector (pCDNA3.1) as described before. After 24 h, both control and lncRNA-transfected cells were seeded in each chamber at a density of 3 × 10 ^5^ cells/reservoir in 70 μL of normal medium for 24 h. The medium was then replaced with fresh medium and the cells were treated with 10 μg/mL of Mitomicyn C (Sigma-Aldrich, St. Louis, MO, USA) for 1 h at 37 °C. After the incubation, the chambers were removed, and cells were further incubated in normal medium. Cells were photographed (1:1 magnification) from T_0_ to T_end_ (8 h for PEA1, 32 h for KURAMOCHI, and 8 h for SKOV3) at regular intervals (2 h each for PEA1 and SKOV3 and 8 h for KURAMOCHI), and the distance covered by cells within a defined area in the gap was measured using NIH ImageJ software (rsb.info.nih.gov/ij). The rate for each time point was calculated for HAND2-AS1 and control transfected, and the results are the average of 3 experiments plotted using GraphPad Prism software Version 7.0a (GraphPad Software, Inc., San Diego, CA, USA).

### 4.9. Adhesion Assay

Coverslips were coated with Fibronectin (10 µg/mL; Calbiochem, San Diego, CA, USA) or Collagen I (10 μg/mL; Invitrogen, Carlsbad, CA, USA) in PBS 1X for 1 h at 37 °C. SKOV3, KURAMOCHI, and PEA1 cell lines were transfected with control pCDNA3.1 and HAND2-AS1 vectors as described before. After 48 h, 40 × 10 ^3^ of control and lncRNA-transfected cells were plated on the top of coated coverslips in triplicates for 2 h at 37 °C. After incubation, the coverslips were washed with Phosphate-Buffered Saline 1X (Euroclone S.P.S., Pero, Italy), fixed in 4% paraformaldehyde (Sigma-Aldrich, St. Louis, MO, USA) for 10 min, and stained with Hoechst (Sigma-Aldrich, St. Louis, MO, USA). The experiment was repeated three times (n = 3) for each cell line. Images were acquired using a confocal microscope (ZEISS LSM 700). For each coverslip, 10 images were acquired and analyzed using ImageJ software (rsb.info.nih.gov/ij). The results plotted are the average of 3 experiments with values normalized and transformed using the formula Y = log(Y) using GraphPad Prism software.

### 4.10. Viability Assay

To assess the viability, SKOV3, KURAMOCHI, and PEA1 cells were transfected with both control pCDNA3.1and HAND2-AS1 vectors. Twenty-four hours after transfection, 1 × 10 ^4^ cells per sample were plated in triplicates on 96-well plates under regular culture conditions. Cell viability was detected 24, 48, and 72 h later using the MTS reagent (Promega, Madison, WI, USA) and represented as T24, T48, and T72. The viability ratio of cells grown in the two different wells was calculated using OD_sample well_/OD_control well,_ where the control well was that with the control vector at time T0.

### 4.11. HDAC Inhibitor Treatment

PEA1 and KURAMOCHI cell lines were treated with Panobinostat (Sigma-Aldrich, St. Louis, MO, USA) along with their respective vehicle control using a final concentration of 10 and 1 nM, respectively. RNA was extracted after 48 h of the treatment. Before performing the Panobinostat treatment experiments, we treated the cell lines with different concentrations, identified the IC50 of the respective cell lines, and used the most suitable dose for the experiments.

### 4.12. mRNA Network

mRNAs significantly correlated with HAND2-AS1 from 489 TCGA-OV samples, with positive and negative correlation thresholds of 0.4 and −0.2, respectively, and *p*-value < 0.05, were downloaded from cBioportal (https://www.cbioportal.org/) [29,30].

The positively and negatively correlated genes were then enriched for KEGG and GO biological process terms using ClueGO and CluePedia in Cytoscape V3.7.1 [32].

### 4.13. HAND2-AS1 ceRNA Network Prediction

In total, 374 RNAseq and 489 miRNAseq TCGA-OV primary tumor samples were downloaded and pre-processed using the GDCRNATools R package [33]. 

For the HAND2-AS1 ceRNA network, the combined list of mRNA targets of miRNAs obtained previously from Diana [40] and miRTarBase [41] were used for ceRNA analysis with GDCRNATools. 

lncRNA–miRNA–mRNA interactions with a hyperPValue <0.05 and corPValue <0.05 were used to build the ceRNA network for ovarian cancer. The network was then visualized using Cytoscape V3.7.1 [31] (Figure 6a). The protein coding genes in the ceRNA network were enriched for GO biological process and KEGG pathway terms (Figure 6b,c).

### 4.14. Figure for Graphical Summary

The Biorender app (https://biorender.com) was used to generate Figure 7.

### 4.15. Statistical Analysis

All the appropriate statistical analyses were performed using GraphPad Prism software Version 7.0a (GraphPad Software, Inc., San Diego, CA, USA) considering *p* < 0.05 as statistically significant. All the details of the tests and significance attached to the asterisks have been listed in Supplementary Table S7.

## 5. Conclusions

This study identified bioinformatically and experimentally the tumor-suppressive role of lncRNA HAND2-AS1 in HGSC and demonstrated for the first time its increase in HGSC cells when treated with the chemotherapeutic HDAC inhibitor Panobinostat. Moreover, we proposed a ceRNA network for HAND2-AS1 in HGSC, which has to be further explored.

## Supplementary Materials

The following are available online at https://www.mdpi.com/1422-0067/21/11/4059/s1. Table S1—List of DE lncRNAs in the three datasets. Figure S1— Details regarding the methylation status of HAND2-AS1 promoter. Figure S2—Expression of HAND2-AS1 after transfection with expression vector used in physiological assays. Table S2—List of mRNAs positively and negatively correlated significantly with HAND2-AS1. Table S3—List of GO BPs enriched from positively correlated mRNAs. Table S4—List of GO BPs enriched from negatively correlated mRNAs. Table S5—List of KEGG pathways enriched from positively correlated mRNAs. Table S6—List of miRNAs and target mRNAs associated with HAND2-AS1 in ceRNA network. Table S7—Details regarding tests of significance obtained from the experimental analysis

## Abbreviations

lncRNA	Long non-coding RNA
ncRNA	Non-coding RNA
HGSC	High-Grade Serous Carcinoma
FT	Fallopian Tube
OC	Ovarian Cancer
HAND2-AS1	HAND2 Anti sense RNA 1
5-AZA	5-Aza-2’–deoxycytidine
mRNA	Messenger RNA
ceRNA	Competing endogenous RNA
HDAC	Histone De-Acetylases
miRNA	Micro RNA
GO BP	Gene Ontology Biological Process
KEGG	Kyoto Encyclopedia of Genes and Genomes
TCGA-OV	The Cancer Genome Atlas – Ovarian Cancer
GTEx	Genotype Tissue Expression
GEPIA	Gene Expression Profiling Interactive Analysis
