# Supplementary figures and images for: Long Non-Coding RNA HAND2-AS1 Acts as a Tumor Suppressor in High-Grade Serous Ovarian Carcinoma
Source: Int J Mol Sci. 2020 Jun 5;21(11):4059. doi: 10.3390/ijms21114059 (PMC7312972; doi:10.3390/ijms21114059)

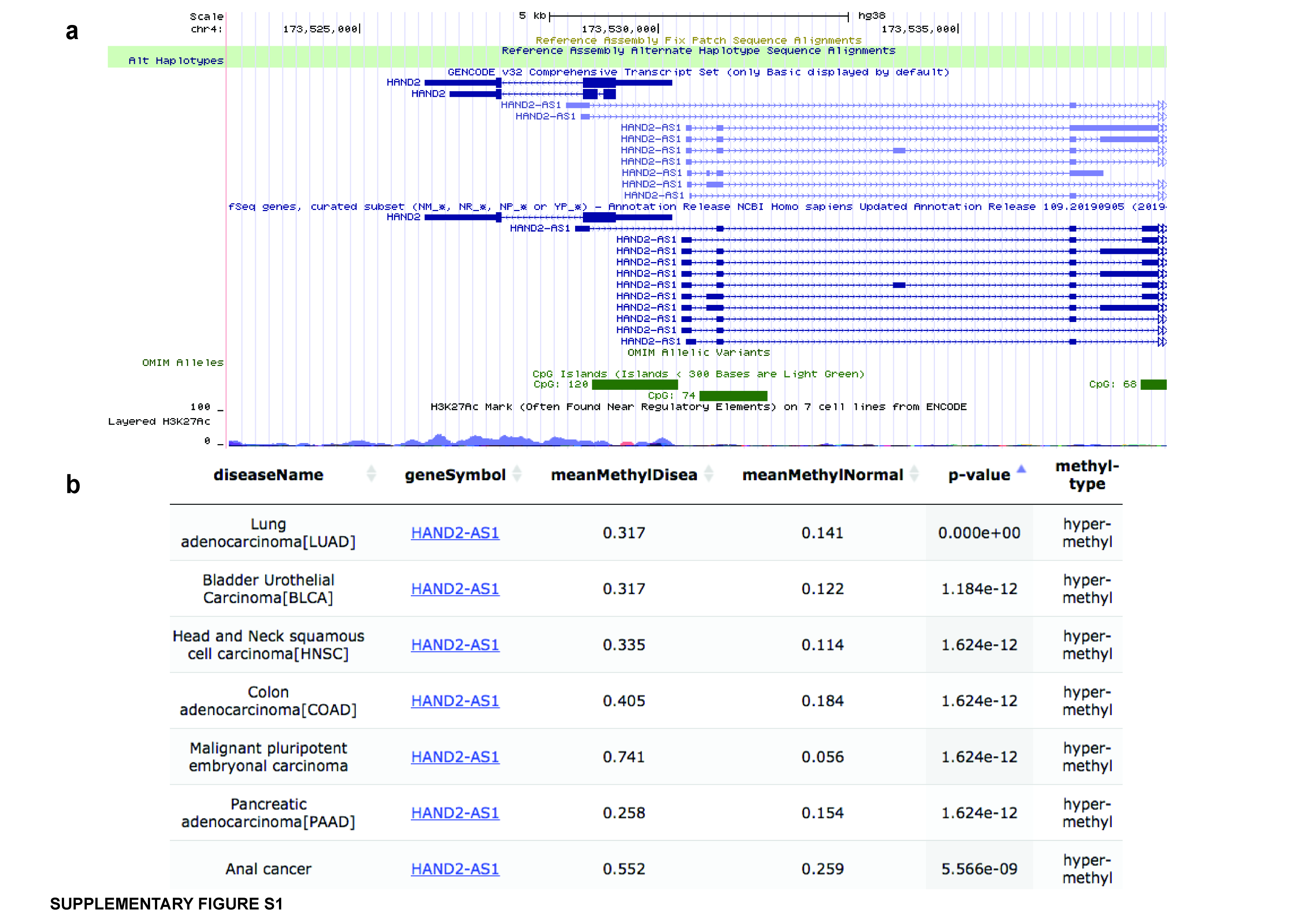

Supplement: Supplementary file 1 [file ijms-21-04059-s001.zip › Supplementary Figure S1.tif]

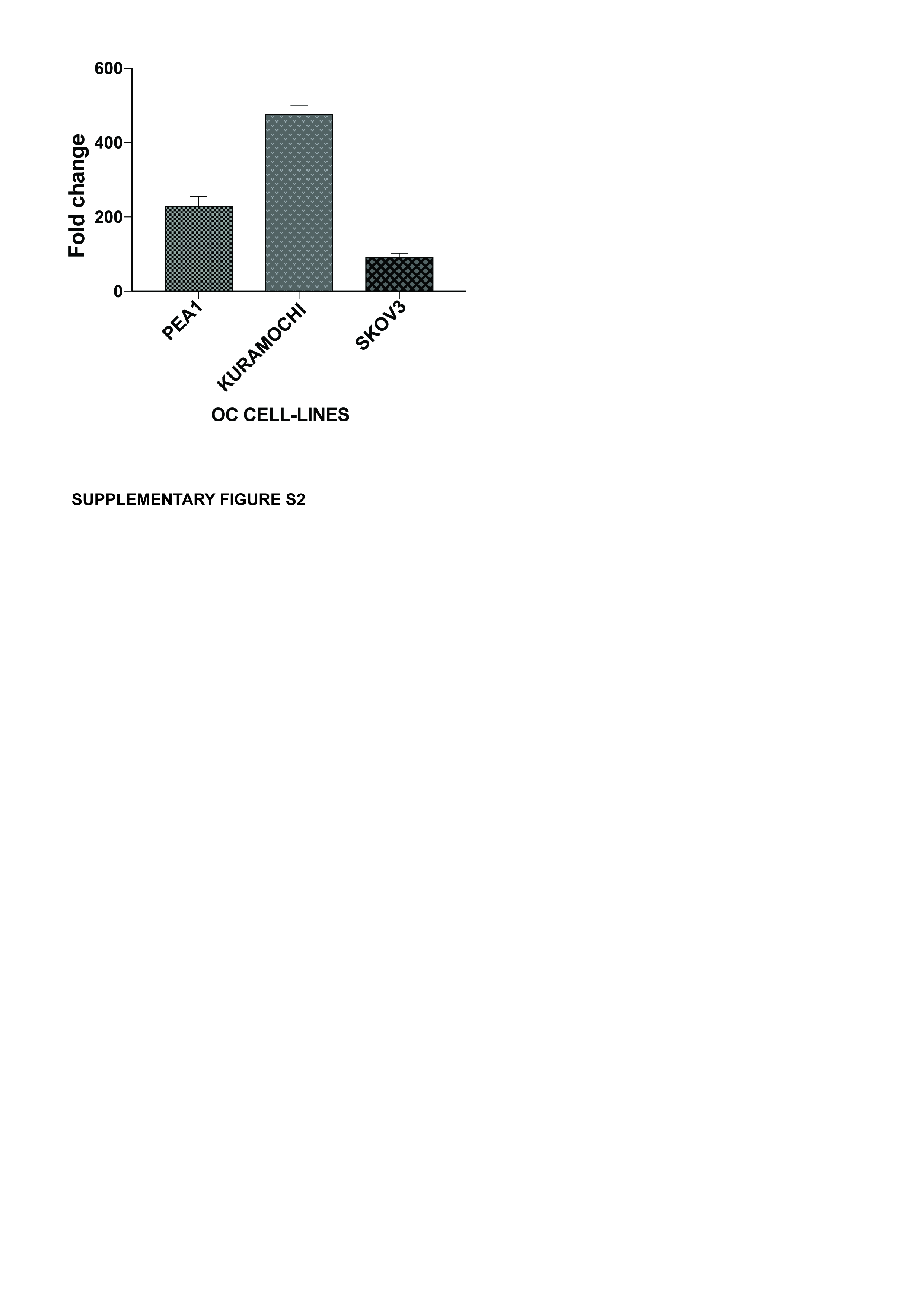

Supplement: Supplementary file 1 [file ijms-21-04059-s001.zip › Supplementary Figure S2.tif]
